# Fracture toughness testing using photogrammetry and digital image correlation

**DOI:** 10.1016/j.mex.2018.09.012

**Published:** 2018-09-29

**Authors:** Wen Hao Kan, Carlos Albino, Daniel Dias-da-Costa, Kevin Dolman, Timothy Lucey, Xinhu Tang, Julie Cairney, Gwénaëlle Proust

**Affiliations:** a Australian Centre for Microscopy and Microanalysis, The University of Sydney, NSW 2006, Australia; b School of Aerospace, Mechanical and Mechatronic Engineering, The University of Sydney, NSW 2006, Australia; c ISISE, University of Coimbra, Rua Luis Reis Santos, 3030-788 Coimbra, Portugal; d School of Civil Engineering, The University of Sydney, NSW 2006, Australia; e Weir Minerals Australia, Artarmon, NSW 2064, Australia

**Keywords:** Digital image correlation with photogrammetry, Digital image correlation (DIC), Photogrammetry, Metal matrix composite, High chromium white cast iron, Fracture toughness

## Abstract

Digital image correlation (DIC) is an optical technique commonly used for measuring displacement fields by tracking artificially applied random speckle patterns, which can sometimes be a problem for tracking small-scale displacements. DIC is particularly useful for tracking the crack mouth opening displacement (CMOD) of a notched metallic specimen subjected to three-point bending for fracture toughness determination because the edges of the notch provide the required textural features for DIC without the need for speckle patterns. This simplifies the set-up process as the specimen and stage geometries do not need to account for the placement of a strain gauge. To enhance the accuracy of DIC, this study then successfully downscaled a photogrammetry technique commonly used to track crack propagation in large scale concrete tests so that the pixel coordinates of the captured images can be automatically related to their real-world coordinates, allowing for small scale displacements to be accurately tracked.

## Highlights

• The CMOD of small three-point bend specimens were tracked using DIC without speckle patterns.
• Accuracy was enhanced by combining a photogrammetry technique with DIC.
• This approach automatically relates the pixel coordinates of the captured images to their real-world coordinates.
• By omitting the need for strain gauges, this approach simplifies the set-up process.

## Graphical abstract

Measuring the crack mouth opening displacement of notched metallic specimens subjected to three-point bending using a combination of photogrammetry and digital image correlation.

**Figure:**
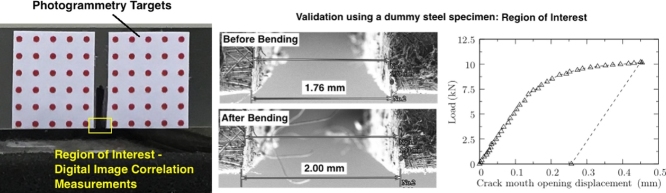

**Specifications Table**

**Table:** 

**Subject Area**	*Engineering*
**More specific subject area:**	Fracture toughness testing, digital image correlation, photogrammetry
**Method name:**	Digital image correlation with photogrammetry
**Name and reference of original method**	This study combines two techniques, Digital Image Correlation (DIC) and Photogrammetry, to track the crack mouth opening displacement (CMOD) of small cast iron composites subjected to three-point bending to determine their fracture toughness. The overall study can be found in [1]. The standard approach to determine fracture toughness using three-point bending can be found in [2]. Rather than using a strain gauge, however, DIC can be used to determine the CMOD, as detailed in [3]. In order to enhance the accuracy of DIC, and to relate pixel coordinates of the captured images to their real-world coordinates automatically, we applied a photogrammetry technique developed by one of our co-authors, which can be found in [4]. *1. Kan, Wen Hao, et al. "Microstructure characterisation and mechanical properties of a functionally-graded NbC/high chromium white cast iron composite." Materials Characterization 136 (2018): 196-205.* *2. ASTM E399, Standard Method of Test for Plane Strain Fracture Toughness of Metallic Materials.* *3. Dai, Xiangjun, et al. "Load capacity evaluated from fracture initiation and onset of rapid propagation for cast iron by digital image correlation." Optics and Lasers in Engineering 51.9 (2013): 1092-1101.* *4. Dias-da-Costa, D., J. Valença, and R. N. F. do Carmo. "Curvature assessment of reinforced concrete beams using photogrammetric techniques." Materials and structures 47.10 (2014): 1745-1760.*
**Resource availability**	–

## Method details

Digital image correlation (DIC) is an optical technique that is commonly used today to perform non-contact measurements of displacement fields [[1], [2], [3], [4], [5]]. Since DIC records displacements across an entire field, the technique is able to obtain heterogenous deformation data, such as localised damage accumulation, crack propagation and stress concentrations, that cannot be otherwise obtained from averaged discrete measurements provided by traditional strain gauges and extensometers without the use of additional (typically expensive) sensors or equipment [1,6]. Outside of simple displacement measurements, DIC techniques can be extremely cost effective as a single DIC package is extremely versatile and can be used for a wide variety of deformation experiments, commercial DIC packages are now widely available, and the costs of digital cameras and computers are increasingly affordable [4].

The premise of DIC lies on the assumption that the characteristics of the surface texture passively follow the deformation of the specimen being analysed and can thus be accurately measured in 2D (or 3D if two cameras are used) by comparing between a series of captured images [[1], [2], [3], [4], [5]]. Therefore, unless the material naturally contains features that can be easily tracked, the first step in the DIC typically involves the application of artificial random speckle patterns [[1], [2], [3], [4], [5]]. After an experiment has concluded and a series of images of the specimen undergoing deformation has been acquired, a DIC algorithm is employed to track displacements between the specimen surface textural features across the images.

Naturally, the accuracy and quality of DIC measurements are deeply affected by the quality of the applied speckle patterns and the image subsets used for correlation [1]. Generally speaking, the image subsets used for these types of analysis should be as small as possible for accurate displacement measurements [3]. This, however, comes into direct conflict with the need for the subsets used to be as large as possible such that the subsets encompass a sufficient amount of distinctive features in the speckle patterns, as is required for reliable DIC [3]. Therefore, the subset sizes required are highly dependent on the quality of the speckle patterns used (such as contrast, speckle sizes and distribution). In practice, spray painting is the preferred method of applying random speckle patterns since this saves a considerable amount of time compared to other more manual approaches and the spray painting patterns can be scaled to ensure that there are sufficient textural details to avoid the subset problem [7]. Nonetheless, since the patterns are still random, the reliability of measuring small amounts of displacement may vary between tests even if the specimens tested and the method of applying the speckle patterns are completely identical. Therefore, it may be advantageous if the use of speckle patterns can be eliminated entirely. If very small-scale displacements are to be accurately measured, it is also worth noting that it is possible to conduct mechanical tests within a scanning electron microscope (SEM) and applying the DIC technique to the captured SEM images to measure relatively large (mm-scale) strain fields [8].

In terms of conventional mechanical testing, the advantages of traditional DIC over the use of strain gauges can be easily highlighted with three-point bending tests of brittle metals, typically to determine fracture toughness values [6]. In these tests, specimens with a fatigued pre-crack notch are loaded in three-point bending such that the notch widens as the load is applied. The widening displacement of the notch, usually referred to as the crack mouth opening displacement or CMOD, is required to compute the fracture toughness value. Since the CMOD is traditionally measured using a strain gauge, it can only provide discrete measurement data of *a single pre-defined location* along the notch [6,7]. In contrast, DIC provides full-field displacement measurements across the entire notch (which allows for more accurate measurements between specimens) and, if desired, DIC can also track and measure the resulting crack propagation when a high speed camera is available [9]. The latter, in particular, can be very difficult (at least when compared to DIC) when using conventional, often expensive sensors [6]. Additionally, since the strain gauge has to maintain stable contact with the notch throughout the test (which can be especially problematic if the specimen size is relatively small), additional provisions, such as the use of bolt-on strain gauges or notches machined with specialised geometries, may need to be employed [10]. Depending on the mechanical testing system available, the use of a strain gauge may also require a specific data logger. Furthermore, the geometry of the stage must also be designed to accommodate the placement of the strain gauge. The non-contact nature of DIC, therefore, simplifies the process considerably while being easy to set-up.

Perhaps the most interesting aspect of using DIC to track the CMOD of brittle metallic specimens subjected to three-point bending is the fact that the notch edges (which are the regions of interest (ROI) when measuring the CMOD) should naturally provide sufficient detail for DIC tracking, regardless of whether the material itself contains sufficient natural textural features. In place of speckle patterns, other optical techniques that utilise the specimen surface can then be simultaneously applied to enhance the accuracy of DIC. One method is to combine DIC with photogrammetry [[11], [12], [13]], the latter being a technique that uses a series of images captured prior to mechanical testing to calibrate the coordinates and boundaries of the measured object, typically with the use of pre-defined patterns. In this context, however, it should be noted that DIC is used as a global measurement tool and photogrammetry is used for scaling to real-world coordinates. If accurate full-field strain measurements around the crack tip are desired in addition to the CMOD, the proposed photogrammetry approach can still be combined with DIC by applying random speckle patterns around the crack tip. Naturally, the inherent issues regarding the use of speckle patterns will still be present to some degree, as were mentioned prior.

This study, therefore combines the photogrammetric technique developed by Dias-da-Costa and Valença et al. [[14], [15], [16], [17]] with DIC to accurately measure the CMOD of a small metallic specimen subjected to three-point bending without strain gauges, thus simplifying the testing procedure. The proposed technique involves the application of circular photogrammetric targets on the surface being tested to accurately and automatically relate real-world coordinates of the displacement field to pixel coordinates. This can be easily accomplished since the real-world distance between each of these targets is known (or can be measured) and a simple algorithm can be used to automatically locate them within an image, which then allows the pixel displacements of the CMOD (as measured by DIC) to be accurately related to its real-world displacements. Since this photogrammetric technique has only been used for large scale testing of concrete beams, this study aims to validate its applicability to a significantly smaller scale.

## Experimental procedure

### Image acquisition and photogrammetry

**Fig. 1:**
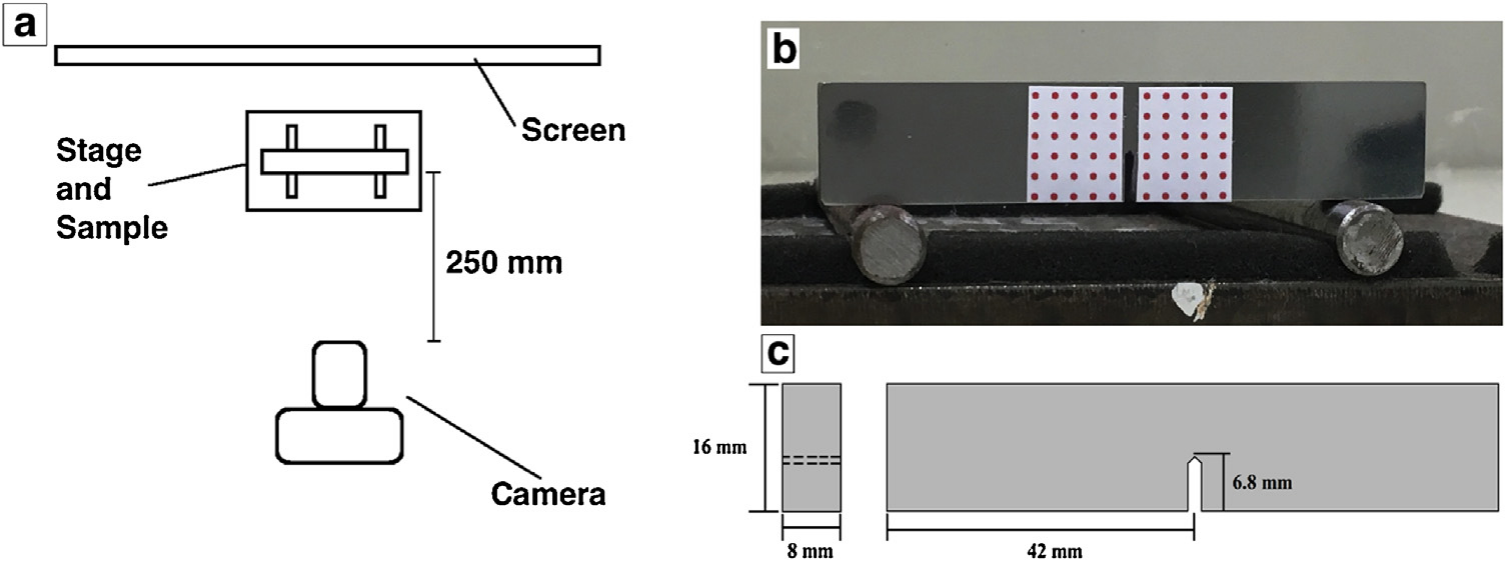
Typical three-point bending set-up: (a) schematic of the set-up, (b) actual specimen with attached photogrammetric targets, (c) schematic of the specimens showing dimensions.

The proposed DIC and photogrammetry technique was employed to measure the CMOD of high chromium white cast iron alloys that have been reinforced with niobium carbides (thus forming a metal matrix composite) while being subjected to three-point bending. By plotting the CMOD with the load data, and assuming that the deformation up to failure is almost entirely linear-elastic, the plane-strain fracture toughness can then be obtained through a series of equations as specified by ASTM E399 [10]. In summary, this mechanical test requires the fabrication of fatigue pre-cracked notched specimens. A detailed study of the investigated material is reported elsewhere [18], but all relevant information pertaining to this study (namely, a summary of the material and the three-point bending test procedures and results) will be detailed here. The schematic of the three-point bending set-up is shown in Fig. 1a. Prior to every three-point bending test, photogrammetric targets were printed on adhesive paper and attached on the fatigue pre-cracked notch specimen as shown in Fig. 1b, with a schematic diagram showing the dimensions of these specimens in Fig. 1c. These targets represent the overall displacement field that was being monitored throughout the test.

Image acquisition was carried out by using a Nikon D810 36.3 M P camera installed on a tripod and equipped with AF-S Micro-Nikor 105 mm f/2.8 G IF-ED macro lenses. The camera was triggered remotely to avoid external vibrations that could impact the measurements and was calibrated beforehand using the toolbox from J. Bouguet [19]. This provided the set of intrinsic parameters that characterise optical, geometric and digital characteristics of the camera. The camera was positioned approximately 250 mm away from the test specimen and a white screen was placed behind the entire set to prevent any background activity from interfering with the images captured (all of which are illustrated in the schematic shown in Fig. 1a). The targets were 900 μm in diameter and distributed over a grid with 2.7 mm pitch in the x- and y- directions, on either side of the notch.

In establishing the relation between world and camera systems, the photogrammetric technique described in studies by Dias-da-Costa et al. [14,15] in the scope of full-field measurement of displacements and strains was used, with the main aspects and equations recovered herein. In particular, this approach assumes that targets are located along a plane that is defined by the surface of the specimen. This assumption has the advantage of requiring a single camera to monitor the specimen behaviour, which further simplifies the analysis. Accordingly, a target on the world plane is projected onto the image plane using the following equation [20]:

(1) X=ωHx ⇔ XYT= ωh1h2h3h4h5h6h7h8h9xy1

where **X** = *(X, Y, T)^T^* are the world plane coordinates, **x** = (*x, y,* 1)*^T^* are the coordinates in the image, *ω* is the scale factor and **H** is a 3 × 3 matrix containing the homographic parameters. The third coordinate 'T' represents the depth of any point located along the ray of projection (between the camera and the test sample) that is being mapped onto the image.

Eq. (1) can also be rearranged in the following equivalent form:

(2) ωT= 1h7x+h8y + h9 h1x+h2y + h3-Xh7x+h8y + h9=0 h4x+h5y + h6-Yh7x+h8y + h9=0

In Eq. (2), there are nine unknowns: the homographic parameters and the ratio *ω / T*, which is constant for each target and is a function of the same homographic parameters. This number can be reduced to eight since the scale factor can be obtained using any known world distance.

Before starting each experiment, the real world coordinates for each circular target were known using the pre-defined dimensions of the grid, which were measured using a Neoscope Tabletop SEM. The corresponding pixel coordinates of each target (i.e. the coordinates within the image) were obtained using the Hough transform algorithm to localise the centre of each target in the complete set of images [21]. Ten images were acquired before starting each bending test to compute the homographic parameters [16,22,23]. For this purpose, the last two expressions in Eq. (2) were applied to each target, such that the following system of equations could be written (where ‘n’ is the number of targets):

(3) $$Wh=\;\left(\begin{array}{ccccccccc} x_{1} & y_{1} & 1 & 0 & 0 & 0 & {-x_{1}X}_{1} & {-y_{1}X}_{1} & {-X}_{1}\\ 0 & 0 & 0 & x_{1} & y_{1} & 1 & {-x_{1}Y}_{1} & {-y_{1}Y}_{1} & {-Y}_{1}\\ x_{2} & y_{2} & 1 & 0 & 0 & 0 & {-x_{2}X}_{2} & {-y_{2}X}_{2} & {-X}_{2}\\ 0 & 0 & 0 & x_{2} & y_{2} & 1 & {-x_{2}Y}_{2} & {-y_{2}Y}_{2} & {-Y}_{2}\\ \vdots & \vdots & \vdots & \vdots & \vdots & \vdots & \vdots & \vdots & \vdots \\ x_{n} & y_{n} & 1 & 0 & 0 & 0 & {-x_{n}X}_{n} & {-y_{n}X}_{n} & {-X}_{n}\\ 0 & 0 & 0 & x_{n} & y_{n} & 1 & {-x_{n}Y}_{n} & {-y_{n}Y}_{n} & {-Y}_{n} \end{array}\right)\left(\begin{array}{c} h_{1}\\ h_{2}\\ h_{3}\\ h_{4}\\ h_{5}\\ h_{6}\\ h_{7}\\ h_{8}\\ h_{9} \end{array}\right)=0$$

Since the number of targets was higher than four, the system of equations was over-determined and the parameters were searched by extracting the eigenvector corresponding to the smallest eigenvalue of W*^T^*W [20].

It should be noted that once the pixel coordinates of each target is calibrated with its real-world coordinates, rigid body motion and any other motion within the initial surface plane are fully accounted for. Since the specimen geometry for this study is designed for plane fracture conditions, out-of-plane motion is very unlikely to occur and therefore, we can assume that all relevant displacements will occur within the initial surface plane and will be fully accounted for by the proposed bi-dimensional technique.

### Digital image correlation

It should be highlighted that after all homographic parameters were retrieved for each specimen, they remain unchanged for any subsequent stage of the test. This allows the use of Eq. (1) to calculate the real coordinates of any given pixel inside the surface plane. It is exactly here that the DIC procedure can be used to track selected critical points. The fact that both vertices defining the mouth notch represent a geometric distinctive feature, they can be tracked automatically using the procedure described here. In particular, this can be done without the need for special painting procedures based on the random speckle patterns usually associated with DIC and often mentioned as a hindrance of the approach [24,25]. In other words, the photogrammetric technique mentioned previously was used to calculate the spatial coordinates for the opposite edges of the notch and determine the CMOD. DIC was used to correlate relative changes in displacements and track down the position of the CMOD edges in the image as it widens.

**Fig. 2:**
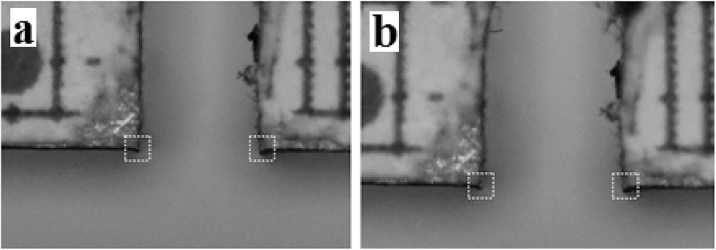
Representation of the ROI: (a) initial reference image before deformation; (b) stage monitored after significant displacements occurred.

The CMOD of each specimen was monitored by tracking the two opposite edges of the notch on images acquired progressively during the test. A square-shaped ROI was defined and centred around each edge of the notch on the initial image taken, as shown in Fig. 2a. This region was then localised in any subsequent images by searching the maximum value for the correlation coefficient given by:

(4) $$r\;=\;\frac{\sum_{m}\sum_{n}{(I}_{mn}-\;\bar{I}\;)(F_{mn}-\;\bar{F}\;)}{\sqrt{[\sum_{m}\sum_{n}{(I}_{mn}-\;\bar{I}\;)2][\sum_{m}\sum_{n}(F_{mn}-\;\bar{F}\;)2]}}$$

where *I* and *F* are the matrices containing the grey values for each pixel inside the ROI in both the initial and final images, respectively, and $\bar{I}$ and $\bar{F}$ are their respective mean values. Once the coordinates (in pixels) for both edges were found, Eq. (1) was then used to calculate their real coordinates and the CMOD was obtained directly by recording the distance between them throughout the test.

### Material information and three-point bending tests

Traditional high chromium white cast irons are alloys that typically contain a significant volume fraction of chromium carbides to improve wear performance at the expense of fracture toughness. The material investigated in this study is a high chromium white cast iron alloy that was further reinforced with niobium carbide. Centrifugal casting was used to the segregate niobium carbide particles toward the outer periphery of the casting in order to create a functionally graded material where the outer surface was harder and more wear resistant (herein referred to as the hard layer) while the bulk material was softer and tougher [18]. As such, the three-point bend specimens were extracted from these two distinct regions of this functionally graded casting, as was reported in the quoted study. Since the microstructures of the specimens are known, we can predict the expected fracture toughness values since there is usually an inverse relationship between fracture toughness and the volume fraction of carbide phase(s) present in high chromium white cast irons [26].

In general, depending on the volume fraction of chromium carbides present, high chromium white cast iron alloys tested in accordance to ASTM E399 can be expected to have K_IC_ fracture toughness values ranging from approximately 20 MPa $\sqrt{m}$ when the carbide volume fraction exceeds 40 vol%, to approximately 30 MPa $\sqrt{m}$ when the carbide volume fraction is decreased to 15 vol% or less [26]. Thus, if the proposed DIC and photogrammetric technique is accurate, the computed fracture toughness values of the composite should fall within this range. However, in terms of the performance of the proposed composite over traditional alloys, since the total carbide volume fractions (niobium carbide and chromium carbide) of the tested specimens obtained from the bulk material and the hard layer are 20 and 39 vol% respectively, they must be benchmarked against traditional high chromium white cast iron alloys that contain 20 and 39 vol% chromium carbides respectively, as is highlighted in Table 1.

**Table 1:** Summary of the microstructures of the two regions of a functionally graded high chromium white cast iron composite from which the three-point bending specimens were obtained, and the corresponding expected fracture toughness as predicted based on the microstructure [26].

	Niobium Carbide Vol%	Chromium Carbide Vol%	Total Carbide Vol%	Metal Matrix Phases	Expected Fracture Toughness K_IC_, (MPa$\sqrt{m}$)
**Hard Layer** **Bulk Material**	27 6	12 14	39 20	Austenite + Martensite Austenite + Martensite	21 – 24 25 – 31

Fatigue pre-cracking on these specimens were done using a servo-hydraulic Dynamic Testing Instron 8501 machine at a frequency of 5 Hz and a load cycle that ranges from 200 N to 2000 N in compression. Crack propagation was monitored using an optical microscope that was positioned such that the top of the notch was in focus. The actual average lengths of the fatigue pre-cracks (required for the determination of the plane-strain fracture toughness) were measured by analysing the fracture surfaces after the tests using an SEM. The three-point bending tests were performed on the specimens using a servo-hydraulic MTS Criterion^™^ Model 43 system by applying a displacement control rate of 0.2 mm/min. Each specimen was loaded such that bending occurred over a span of 64 mm. The plane-strain fracture toughness values, or K_Ic_, were calculated using the equations specified in ASTM E399 where the dimensions of the specimens, the notch lengths, the CMOD, failure loads and the fatigue pre-crack lengths are all used for the computations [10].

### Validation

Prior to the application of the proposed DIC and photogrammetry technique to actual three-point bending tests of the cast iron specimens, the accuracy of the technique was first validated. Since this present technique only tracks the CMOD along the specimen surface exposed to the camera, the use of a strain gauge to validate the technique is not ideal because a strain gauge would only record the average CMOD of the notch across the *thickness* of the sample. Therefore, it was proposed that the best approach to validating the proposed technique would be to measure the actual CMOD of a specimen along the exposed surface of the specimen before *and* after three-point bending and relating the measured CMOD values to those measured via the proposed technique. However, since the brittle cast iron specimens were expected to fail under (or extremely close to) plane-strain conditions, they would most likely experience a sudden fracture shortly after yielding, rendering them unsuitable for post-bending measurements.

Therefore, for validation purposes, a *ductile* steel specimen with the same dimensions of the actual cast iron specimens (though without the fatigue pre-crack) was specially fabricated. This steel specimen would then experience significant plastic deformation after yielding, which would then result in a permanent increase in the CMOD. The CMOD along the exposed surface of the specimen can then be accurately measured using an SEM before and after the test and be compared to the CMOD that was calculated via the proposed DIC and photogrammetry technique. It should be stated that the toughness value of the steel specimen cannot be determined in this way (since the material did not actually fracture under plane-strain conditions), and thus, the CMOD measured in this way was purely for validation purposes.

## Results and discussion

### Validation results

At the start of the validation test, ten initial pictures were taken as described prior. The homographic parameters were then obtained from the mean value of target coordinates detected in the ten initial pictures. This information was used to scale the images and obtain the corresponding real world coordinates, which were then compared with the actual values determined using the known grid size. Any difference between the actual coordinates of each target and the corresponding calculated values is, therefore, an error originating from the homography and scaling procedure. The x- and y-direction distribution of this error, was an average of 9 μm and 8 μm, respectively. This corresponds to an average and root mean square of the distance between exact and calculated coordinate values of 13 μm and 14 μm, respectively. These errors should be viewed in the context of the circular photogrammetric targets used, where each target is 900 μm in diameter and distributed over a 2.7 mm by 2.7 mm grid.

**Fig. 3:**
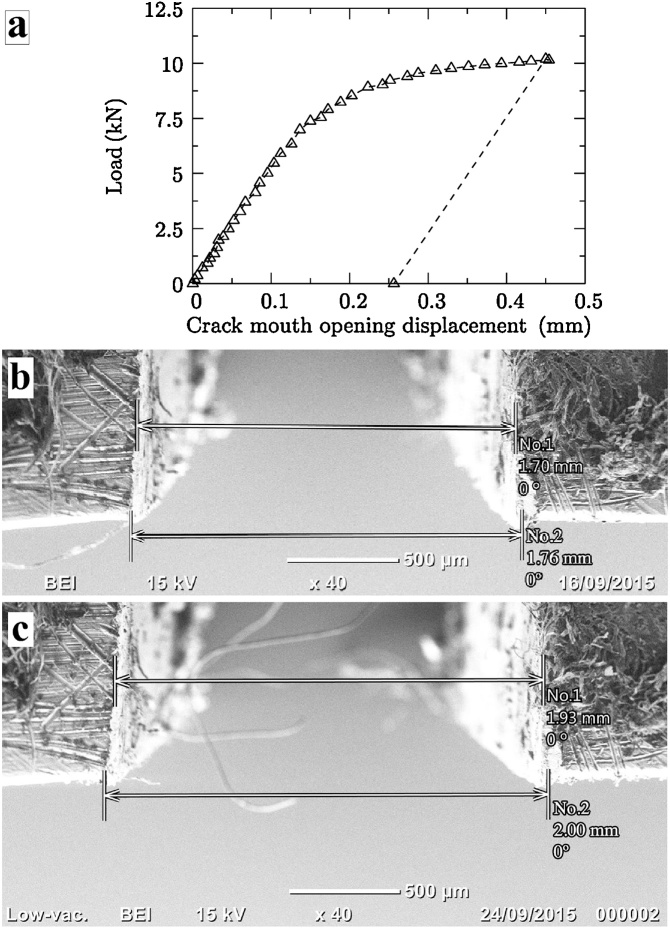
**(a)** CMOD measured during the test, with an onset of plasticity at approximately 6.9 kN and tested up to a load of 10.2 kN; and validation using SEM **(b)** before and **(c)** after the test.

After this step, the image correlation procedure was applied to track the CMOD. It should be mentioned that during the test, images were acquired at intervals of 4 s A rectangular ROI was then designated with 70 pixels and centred around each edge defining the notch on the initial image. For illustration purposes, the representation of the ROIs centred on each edge before and after significant displacements have occurred has been shown previously in Fig. 2, whereas Fig. 3a shows the result obtained for all monitored stages during the test up to a load of 10.16 kN. There was a relatively smooth evolution of the CMOD measurements with loading, which highlighted the stability of the technique and the ability to measure relatively small displacements. In terms of the overall behaviour, the linear elastic regime extends up to a CMOD of 0.15 mm, then the steel begins to bend plastically. Finally when unloaded at a CMOD of 0.45 mm, the specimen recovers as a function of its initial stiffness, as expected of steels. The final CMOD measured by the proposed technique was approximately 0.257 mm upon unloading.

The CMODs of the dummy specimen as measured using the Neoscope Tabletop SEM before and after three-point bending are 1.76 mm and 2.00 mm respectively, as are also shown in Fig. 3b and c respectively. Thus, the overall CMOD as measured using the SEM was found to be 0.24 mm, which is in close agreement with the CMOD of 0.257 mm obtained via image processing, a difference of approximately 7%. This discrepancy can also be attributed to spatial distortion that typically occurs in SEMs at very low magnifications [27,28].

### Three-point bending of the cast iron composites

**Fig. 4:**
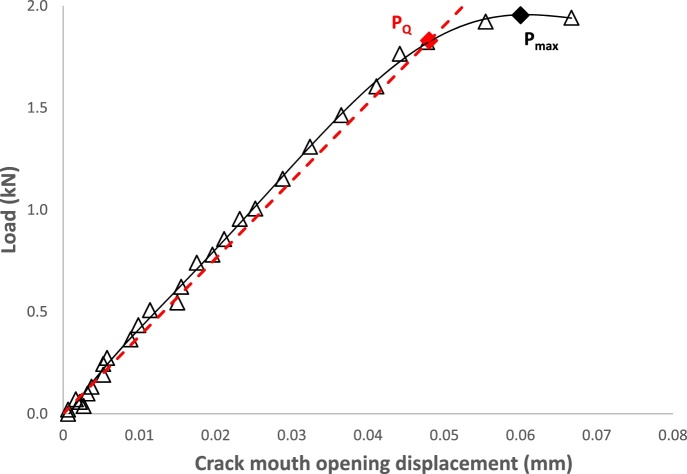
Typical Load vs. CMOD data obtained using the proposed image processing technique. The extrapolation of results in between points is shown by the black line. 95% secant slope shown by the red dashed line. P_Q_ and P_max_ values are marked on the plot.

Once the reliability of the proposed technique has been validated, the actual three-point bending fracture toughness tests of the cast iron composites were conducted. The main differences between the load vs. CMOD curves of the cast iron composites and the dummy steel specimen are that the loads at failure are considerably lower, and that most of the deformation that occurred prior to failure was linear-elastic. The two differences were due to the fact that the cast iron composite specimens were fatigue pre-cracked and were also significantly more brittle (due to the presence of a high volume fraction of ceramic phases). This can be observed with the typical load vs. CMOD graph as shown in Fig. 4. As each data point on the graph is separated by an interval of 4 s, data between points would have to be extrapolated. This is relevant for the determination of the linear elastic plane-strain failure load P_Q_ which is obtained by drawing a line representing a 95% secant slope (see the red dashed line in Fig. 4) from the linear portion of the graph and recording the load at which both lines intercept.

Furthermore, the load vs. CMOD graph is also used for determining if the material failed under plain-strain conditions. This is done by dividing the maximum load (P_max_) with P_Q_, and if the value exceeds 1.10 [10], some amount of plastic deformation has occurred ahead of the crack tip. Using the data collected from the image processing technique, the calculated K_Ic_ value and the plane-strain assessment of each test is shown in Table 2. As can be observed, the hard layer failed under plane-strain conditions while the bulk material experienced a very small degree of plastic deformation as the P_max_/P_Q_ values very slightly exceeded 1.10. For comparative purposes, and since the exceedance was very small, we shall assume that the bulk material specimens failed under plane-strain conditions. The average K_Ic_ values for the hard layer and the bulk material are 20.8 MPa $\sqrt{m}$ and 26.7 MPa $\sqrt{m}$ respectively, with standard deviations of 2.7% and 4.0%, respectively.

**Table 2:** Results from each three-point bending fracture toughness test.

Test No.	Region	Linear Elastic Plane Strain Failure Load, P_Q_ (N)	Maximum Load, P_max_ (N)	Plane-strain Assessment (P_max_/P_Q_)	Fracture Toughness, K_Ic_ (MPa$\sqrt{m})$
1	Hard Layer	2013	2055	1.02	21.6
2	Hard Layer	1675	1847	1.10	20.6
3	Hard Layer	1825	1954	1.07	20.3
4	Bulk Material	2550	2842	1.11	28.2
5	Bulk Material	2250	2509	1.12	26.3
6	Bulk Material	2325	2704	1.16	25.7

A comparison between the expected fracture toughness results in Table 1 and the actual fracture toughness results reported in Table 2 shows that the hard layer performed below expectations while the bulk material performed within the expected range. A detailed explanation for this, based on detailed fracture surface analysis and microstructural characterisation, is also provided in the study of the parent material from which the specimens were obtained from [18]. What is important, however, is that the fracture toughness results of both the hard layer and the bulk material, as calculated using the proposed DIC and photogrammetry technique, were consistent with those of traditional high chromium white cast iron alloys (20 MPa $\sqrt{m}$ to 33 MPa $\sqrt{m}$ as reported earlier). This further validates the use of photogrammetry in conjunction with DIC for micron-scale CMOD measurements.

### Technique refinement

The suitability of the proposed photogrammetry and DIC technique for the measurement of micron-scale deformations using low-cost cameras, at least in the context of three-point bending, has been shown both through a specific validation experiment and with the obtained fracture toughness results of the composites being tested. Three-point bending specimens are particularly unique because the edges of the notch provide the features required for DIC tracking, and therefore, speckle patterns are not at all required. Photogrammetry was then successfully and accurately used to correlate the real-world coordinates to camera coordinates. Therefore, the combination of both photogrammetry and DIC was shown to be very good complements to each other.

The photogrammetry technique used in this study, which as mentioned prior was developed by Dias-da-Costa et al. for the investigation of crack propagation in concrete beams [[14], [15], [16]], was found to also be sufficiently reliable for use with significantly smaller specimens and displacements. The main challenge to this approach is naturally the size of the photogrammetric targets relative to the displacement field being investigated. For instance, the diameter of the circular targets used in monitoring large-scale concrete beams can be as large as 10 mm. As the circularity of the targets is an important criterion for the proposed photogrammetry technique, it is far easier to apply photogrammetry targets that have near-perfect circularity if the diameters are larger. In this study, the diameter of the targets was 900 μm, which was still suitable for the technique. However, since the ROIs in this study are just the surrounding regions close to the notch edges, increased accuracy could result if the diameter of the targets can be further reduced, thus resulting in a smaller grid but with the same (or greater) number of targets. The challenge here would then be the maintenance of circularity as the size of the targets is reduced.

One other challenge, which also applies to any traditional DIC technique involving a camera, is the sampling rate. In this study, images were captured every 4 s, which was found not to be a critical issue during linear-elastic deformation, but required a greater degree of extrapolation during the onset of plasticity just prior to failure. Naturally, a faster image acquisition rate is desired, but rapid image acquisitions could result in unintended vibrations that could affect the quality of the images captured. Granted, this might be a camera limitation, but is a problem nonetheless especially when analysing small specimens where deformations are occurring on the micron-scale. This argument is a valid critique of using low-cost cameras for DIC, though the benefits of using DIC outweigh those of traditional strain gauges. If a rapid sampling rate is critical and tracking the rate of crack propagation is needed, then a high speed camera can be used [9]. As far as this study is concerned, however, the combination of photogrammetry and DIC is the innovation that is being proposed and the sampling rate, being a critique of general DIC over strain gauges, is outside the scope of this study.

In fact, the numerous benefits of using a non-contact optical system over traditional strain gauges to track the CMOD were indeed as described by Dai et al. [6]. Firstly, a strain gauge is only able to measure the CMOD across a single pre-defined location on the notch while a DIC technique allows the CMOD to be measured across the entire notch. Therefore, if a strain gauge is used, additional provisions (such as bolt-on strain gauges or introducing additional geometries into the notches) have to be taken for the placement of the strain gauge, and even a specialised stage may be required simply to accommodate the strain gauge [10]. A non-contact CMOD measurement method, such as the one proposed in this study, completely eliminated the need for those provisions. The only set-up required for our proposed method were the attachment/application of the photogrammetric targets, camera placement/focus, and if required, a light source. Secondly, the DIC technique allowed the deformation fields of the specimens to be analysed after the tests have concluded, which if desired, could yield valuable information such as crack initiation time and crack propagation rate.

## Conclusion

In this study, the authors presented an innovative non-contact technique based on the combination of photogrammetry and digital image correlation to automatically monitor the CMOD of specimens in the scope of three-point bending fracture toughness testing. This technique was first validated using a dummy steel specimen, where it was shown to be fully capable of accurately tracking the CMOD of the notch. Unlike conventional contact-based measurement techniques, the proposed technique allows the CMOD to be tracked across any location of the notch, and without the need for particular load stages and specific strain gauges adapted to the notch of the specimen. Also, due to the combination of photogrammetry and DIC, there was no difficulty in regards to the preparation of the surface, other than attaching a grid of circular targets. Since random speckle patterns were not needed, the proposed technique was an advantage over commercial DIC packages as the use of speckle patterns is particularly challenging when attempting to measure small changes in displacements in comparatively small specimens using low-cost cameras. Instead, both vertices defining the notch mouth already contained distinctive features that was easily and automatically tracked during the experiments. In addition, although not the purpose of this article, crack propagation and strain fields around the top of the crack tip could also be tracked and analysed using this technique as an extra benefit. If K_Ic_ is the only result of interest, then the accuracy of this technique will only depend on the extrapolation involved in using the load vs. CMOD curve in determining the plane strain failure load since each point on the graph is scattered depending on the image acquisition rate (4 s in this case). This rate can be improved if necessary depending on the camera limitations, but this is a critique of DIC in general, not of the proposal to combine photogrammetry with DIC. One other drawback of the method is the fact that the CMOD was only tracked along the surface of the specimen, disregarding the third-dimension. However, this was not critical as the specimen was loaded using a plane-strain set-up.

## Conflict of interest

The authors have declared that there is no conflict of interest.
